# Associations between OGTT results during pregnancy and offspring TSH levels: a birth cohort study

**DOI:** 10.1186/s12884-024-06554-4

**Published:** 2024-05-17

**Authors:** Meng Yang, Zhongqiang Cao, Wanting Zhu, Xiaoyuan Feng, Jieqiong Zhou, Jiuying Liu, Yuanyuan Zhong, Yan Zhou, Hong Mei, Xiaonan Cai, Liqin Hu, Aifen Zhou, Han Xiao

**Affiliations:** 1 Institute of Maternal and Child Health, Tongji Medical College, Wuhan Children’s Hospital (Wuhan Maternal and Child Health care Hospital, Huazhong University of Science and Technology, Wuhan, 430000 China; 2 Department of Obstetrics, Tongji Medical College, Wuhan Children’s Hospital (Wuhan Maternal and Child Health care Hospital, Huazhong University of Science and Technology, Wuhan, China; 3 Department of echocardiography, Tongji Medical College, Wuhan Children’s Hospital (Wuhan Maternal and Child Health care Hospital, Huazhong University of Science and Technology, Wuhan, China

**Keywords:** Gestational diabetes, Oral glucose tolerance test, Congenital hypothyroidism, Birth cohort study

## Abstract

**Background:**

Limited evidence exists regarding the association between gestational diabetes mellitus (GDM) and elevated levels of thyroid-stimulating hormone (TSH) in newborns. Therefore, this study aimed to investigate the potential risk of elevated TSH levels in infants exposed to maternal GDM, considering the type and number of abnormal values obtained from the 75-gram oral glucose tolerance test (OGTT).

**Methods:**

A population-based, prospective birth cohort study was conducted in Wuhan, China. The study included women who underwent GDM screening using a 75-g OGTT. Neonatal TSH levels were measured via a time-resolved immunofluorescence assay. We estimated and stratified the overall risk (adjusted Risk Ratio [RR]) of elevated TSH levels (defined as TSH > 10 mIU/L or > 20 mIU/L) in offspring based on the type and number of abnormal OGTT values.

**Results:**

Out of 15,236 eligible mother-offspring pairs, 11.5% (1,753) of mothers were diagnosed with GDM. Offspring born to women diagnosed with GDM demonstrated a statistically significant elevation in TSH levels when compared to offspring of non-GDM mothers, with a mean difference of 0.20 [95% CI: 0.04–0.36]. The incidence of elevated TSH levels (TSH > 10 mIU/L) in offspring of non-GDM women was 6.3 per 1,000 live births. Newborns exposed to mothers with three abnormal OGTT values displayed an almost five-fold increased risk of elevated TSH levels (adjusted RR 4.77 [95% CI 1.64–13.96]). Maternal fasting blood glucose was independently and positively correlated with neonatal TSH levels and elevated TSH status (TSH > 20 mIU/L).

**Conclusions:**

For newborns of women with GDM, personalized risk assessment for elevated TSH levels can be predicated on the type and number of abnormal OGTT values. Furthermore, fasting blood glucose emerges as a critical predictive marker for elevated neonatal TSH status.

**Supplementary Information:**

The online version contains supplementary material available at 10.1186/s12884-024-06554-4.

## Background

Congenital hypothyroidism (CH), a neonatal metabolic disorder marked by inadequate thyroid hormone synthesis, which is a major metabolic anomaly in newborns with potential irreversible neurodevelopmental impacts if undiagnosed [1, 2]. Newborn screening protocols targeting thyroid-stimulating hormone (TSH) levels have been effective in early CH detection [3–5]. Elevated TSH levels in neonates are linked to detrimental effects on brain and neurological development [6]. Identifying early indicators of elevated TSH in newborns is crucial for reducing the global health burden of CH.

Gestational diabetes mellitus (GDM), characterized by glucose intolerance during pregnancy, is associated with adverse perinatal outcomes like neonatal hypoglycemia and respiratory distress [7–9]. Studies suggest a correlation between GDM and elevated TSH levels in newborns, but research in this area is limited by outdated publication dates, small sample sizes, case-control study designs and inadequate control for potential confounding factors [10, 11]. Additionally, the use of a GDM diagnosis alone as the primary factor in studying its link with future disease risks is inadequate, as it doesn’t fully account for the severity or specific characteristics of maternal blood sugar abnormalities during pregnancy, like varying fasting or postprandial glucose levels [12]. The oral glucose tolerance test (OGTT) has gained prominence as a tool for predicting maternal and fetal outcomes, proven useful in assessing risks of maternal type 2 diabetes, type 1 diabetes in children, and cognitive decline in the elderly [13–15]. However, the effectiveness of OGTT in specifically predicting the risk of elevated neonatal TSH levels in offspring of women with GDM is still uncertain.

Given the limited and inconsistent literature, a prospective birth cohort study with a detailed assessment of potential confounders was warranted. In the present study, we prospectively investigated the association between the results of OGTT, based on the type and number of abnormal values, and the levels of TSH, as well as the risk of elevated TSH status in neonates born to women diagnosed with GDM. Further, we examined the independent effect of different OGTT values on TSH status.

## Materials and methods

### Study design and participants

This study is based on the prospective birth cohort carried out in Wuhan, China. The cohort design has previously been published elsewhere [16]. A total of 21,472 pregnant women were recruited from September 2012 to October 2019. Each participant was required to complete a structured questionnaire at enrollment and provide urine and blood samples before delivery. Women with missing data for the 75-g 2-hour OGTT (*n* = 5,147), those with pre-existing or prenatal thyroid disorders or medications for thyroid treatment (*n* = 85), individuals with missing demographic data (*n* = 219), and infants with missing neonatal TSH data (*n* = 785) were excluded from the analysis (see Figure S1).

### Diagnosis of GDM

Participants in the study, who did not have a prior history of type 1 or type 2 diabetes, were subjected to a universal GDM screening using a 75-g OGTT conducted between 24 and 28 weeks of gestation. The diagnosis of GDM adhered to the guidelines established by the International Association of Diabetes and Pregnancy Study Groups (IADPSG) [17]. According to these guidelines, the following cutoff values were used for OGTT: fasting ≥5.1 mmol/L (92 mg/dL); 1-hour ≥ 10.0mmol/L (180 mg/dL); 2-hour ≥ 8.5 mmol/L (153 mg/dL). GDM was defined as one or more abnormal OGTT values. None of the pregnant women with GDM in this study received medications or insulin therapy.

### Exposures

The main exposures of interest were the type (fasting, 1-hour, or 2-hour) of the 75-g OGTT and the number of abnormal values detected (zero, one, two, or three) according to the IADPSG criteria.

### TSH status

Newborn screening for CH involves testing the levels of TSH in the heel blood sample taken 48–72 h after birth. Newborn TSH levels were measured using the time-resolved immunofluorescence assay method. However, there is ongoing debate regarding the optimal cutoff values for accurately detecting CH in newborns. Therefore, in our analysis, we defined TSH status based on two threshold levels: TSH levels > 10 mIU/L and > 20 mIU/L [18, 19].

### Covariates

We collected data on maternal demographics, socioeconomic status, and lifestyle characteristics through a structured questionnaire before recruitment. The variables included maternal age, gestational week, educational level (≤9/10–12/13–15/≥16 schooling years), physical activity (Never or rarely, 1–2 days/week, 3–4 days/week, 5–6 days/week, Daily), smoking status (yes/no), alcohol drinking (yes/no), multiparity (yes/no) and gravidity (1/2/≥2). The delivery information was derived from electronic health records, including child sex (male/female), cesarean delivery (yes/no) and birth weight. The pre-pregnancy BMI was determined by dividing the pre-pregnancy weight in kilograms by the square of the height in meters. To calculate gestational weight gain, the weight at the time of admission for delivery is subtracted from the pre-pregnancy weight. The variable adjustment sets were determined based on previous studies [20–22] and a directed acyclic graph (DAG).

### Statistical analysis

Frequencies and descriptive statistics were used to summarize the baseline characteristics of the participants. Mean ± SD was presented for continuous variables with normal distributions, while median and interquartile range (25th–75th percentile) was used for those with skewed distributions. Local regression (LOESS) methods were applied to fit the data and generate spline plots with 95% confidence intervals (CIs) for TSH and TSH status as dependent variables and OGTT values (fasting, 1-hour and 2-hour) as independent variables in an unadjusted analysis. The incidence of elevated TSH status was estimated and further stratified by the type and number of abnormal OGTT values. Linear regression models were utilized to determine the association between abnormal OGTT and offspring TSH levels, and Modified Poisson regression models with robust variance estimates were used to estimate the association between abnormal OGTT and offspring TSH status. A linear trend was tested by treating the ordinal value as a continuous variable in the regression models. All multivariable models were adjusted for maternal age, gestational week, pre-pregnancy BMI, gravidity, weight gain, education level, physical activity, smoking status, drinking status, delivery model, fetal sex and birth weight. Results were presented as adjusted risk ratios (RRs) with 95% CIs and were stratified by the type and number of abnormal OGTT values, with women without GDM as the reference group. To determine the independent association of each OGTT value (fasting, 1-hour, and 2-hour) with offspring TSH and TSH status, we developed a second prediction model that was adjusted for all three OGTT values and the aforementioned covariates.

Several sensitivity analyses were performed. Firstly, women with missing data on selected confounders were included in the study population, and multiple imputation was used to account for the missing data. Secondly, women with selected pregnancy complications during pregnancy were excluded from the analysis. Lastly, logistic regression analysis using odds ratios (ORs) instead of RRs was employed to estimate the association between abnormal OGTT and offspring TSH status, as the incidence of elevated TSH status is relatively infrequent.

All data were analyzed using R version 4.1.0. Statistical significance was determined by a two-sided *P*-value of less than 0.05.

## Results

### Characteristics of the study cohort

The study included 15,236 mother-offspring pairs, among whom 1,753 (11.5%) mothers were diagnosed with GDM. The mean age of mothers at delivery was 28.97 ± 3.62 years, and the mean gestational age was 39.26 ± 1.17 weeks. A comparison of descriptive characteristics between the GDM and NGT groups was conducted, with the results presented in Table 1. These findings indicated significant differences across all variables, with the exception of smoking status, drinking status, physical activity level, fetal sex, and TSH levels, for which no significant differences were observed.

**Table 1:** Characteristics of participants with or without gestational diabetes mellitus (GDM) (*N* = 15,236)

Characteristic	Total (*N* = 15,236)	GDM (*N* = 1,753)	NGT (*N* = 13,483)	*P* value
*Variables in mothers*				
Maternal age at delivery (years)	28.97±3.62	30.29±4.11	28.79±3.51	< 0.001
Gestational week (weeks)	39.26±1.17	38.91±1.22	39.30±1.16	< 0.001
Prepregnancy BMI (kg/m^2^), %				< 0.001
< 18.5	2920 (19.2)	209 (11.9)	2711 (20.1)	
18.5–24	10,232 (67.2)	1111 (63.4)	9121 (67.6)	
24–28	1750 (11.5)	344 (19.6)	1406 (10.4)	
> 28	334 (2.2)	89 (5.1)	245 (1.8)	
Education level (schooling years), %				< 0.001
≤9	1127 (7.4)	173(9.9)	954(7.1)	
10–12	2931 (19.2)	376 (21.4)	2555 (18.9)	
13–15	10,306 (67.6)	1118 (63.8)	9188 (68.1)	
≥16	872 (5.7)	86 (4.9)	786 (5.8)	
Smoking (yes), %	3871 (25.4)	447 (25.5)	3424 (25.4)	0.948
Alcohol drinking (yes), %	352 (2.3)	39 (2.2)	313 (2.3)	0.866
Gestational weight gain at delivery (kg)	16.41 (4.94)	16.56±4.87	15.30±5.38	< 0.001
Multiparity (yes), %	2759 (18.1)	421 (23.92)	2338 (17.3)	< 0.001
Gravidity, %				< 0.001
= 1	8500 (55.8)	809 (46.1)	7691 (57.0)	
= 2	3830 (25.1)	479 (27.3)	3351(24.9)	
> 2	2906 (19.1)	465 (26.5)	2441 (18.1)	
Cesarean delivery (yes), %	9022 (59.2)	1236 (70.5)	7786 (57.7)	< 0.001
Physical activity during pregnancy, %				0.581
Never or rarely	1639 (10.8)	186 (10.6)	1453 (10.8)	
1–2 days/week	1531 (10.0)	171 (9.8)	1360 (10.1)	
3–4 days/week	1052 (6.9)	118 (6.7)	934 (6.9)	
5–6 days/week	302 (2.0)	27 (1.5)	275 (2.0)	
Dalily	10,712 (70.3)	1251 (71.4)	9517 (70.2)	
Fasting glucose (mmol/L)	4.40±0.50	4.99±0.92	4.32±0.35	< 0.001
OGTT1-h (mmol/L)	7.13±1.62	9.33±1.91	6.84±1.33	< 0.001
OGTT2-h (mmol/L)	6.41±1.40	8.30±2.21	6.16±1.02	< 0.001
*Variables in neonates*				
Child sex (male), %	8031 (52.7)	936 (53.4)	7095 (52.6)	0.559
Birth weight (Kg)	3343.75 (418.57)	3.37±0.46	3.34±0.41	0.002
TSH (mIU/L)	2.70 [1.60, 4.03]	2.78 [1.68, 4.08]	2.69 [1.59, 4.02]	0.118

Continuous data are presented as mean ± SD or median with interquartile range

GDM, gestational diabetes mellitus; NGT, normal glucose tolerance; BMI, body mass index; OGTT, oral glucose tolerance test results; TSH, thyroid stimulating hormone

### Effect of abnormal OGTT results during pregnancy on TSH levels in the offspring

**Fig. 1:**
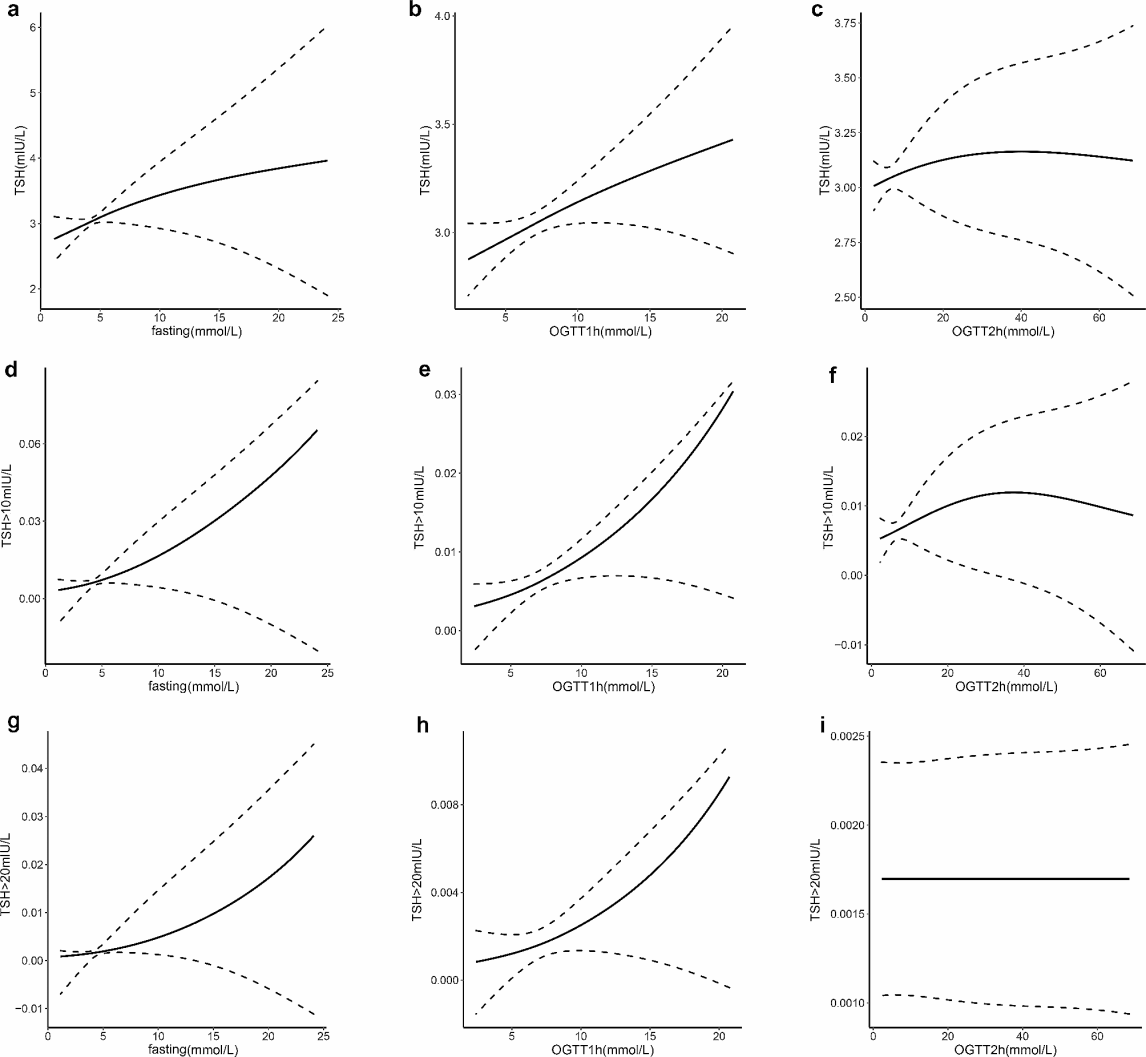
Spline plots showing the TSH levels and TSH status in relation to the fasting (**a, d, g**), 1 h (**b, e, h**) and 2 h (**c, f, i**) of 75-g OGTT values. OGTT, oral glucose tolerance test results; TSH, thyroid stimulating hormone

Among the 1,753 GDM patients, 1,236 (8.1%), 367 (2.4%) and 150 (1.0%) had one, two and three abnormal OGTT values, respectively. Furthermore, 821 (5.4%) had abnormal fasting glucose, 718 (4.7%) had abnormal 1-hour glucose, and 881 (5.8%) had abnormal 2-hour glucose values. The plotted spline curves showed a steady increase in offspring TSH levels as OGTT values elevated, with the most notable increase observed for fasting and 1-hour glucose levels (Fig. 1a and c). These results were supported by both crude and adjusted linear regression analyses (Table S1). After adjusting for confounding factors, newborns of women diagnosed with GDM and one abnormal OGTT value had significantly higher TSH levels compared to those with zero abnormal values (0.20[95% CI 0.04–0.36]; 0.22 [95% CI 0.03–0.41]). Similarly, newborns whose mothers with abnormal fasting glucose had higher TSH levels in both the crude (0.27, 95%CI: 0.04–0.49) and adjusted models (0.29, 95%CI: 0.06–0.52) (Table 2). However, this analysis did not provide independent information between each OGTT value and TSH, as women with more than one abnormal value were included. To explore the independent association between each of the three OGTT values and neonatal TSH, a second prediction model for GDM was developed that included all three OGTT values and additional variables (Table S2). Ultimately, the analysis revealed that only newborns of women with abnormal fasting glucose had higher TSH levels.

**Table 2:** Changes of TSH levels in the offspring by number and type of abnormal OGTT values (*N* = 15,236)

Abnormal OGTT value	*N* (%)	TSH (mIU/L)	
		Crude β (95% CI)	Adjusted β^a^ (95% CI)
*Abnormal values, n*			
0	13,483(88.5)	Reference	Reference
≥ 1 (GDM)	1,753(11.5)	0.15(-0.01,0.31)	**0.20(0.04,0.36)**
1	1,236(8.1)	0.18(-0.01,0.37)	**0.22(0.03,0.41)**
2	367(2.4)	-0.01(-0.35,0.32)	0.05(-0.28,0.39)
3	150(1.0)	0.31(-0.20,0.83)	0.38(-0.14,0.90)^b^
*Abnormal values, type*			
Fasting (≥ 5.1 mmol/L)	821(5.4)	**0.27(0.04–0.49)**	**0.29(0.06,0.52)**
1-hour (≥ 10.0 mmol/L)	718(4.7)	0.13(-0.11,0.37)	0.18(-0.07,0.42)
2-hour (≥ 8.5 mmol/L)	881(5.8)	0.02(-0.19,0.24)	0.09(-0.14,0.31)

Bold font indicates significant association. OGTT, oral glucose tolerance test results; GDM, gestational diabetes mellitus; TSH, thyroid stimulating hormone

^a^Values reflect the results of linear regression model including women without GDM as the reference group (i.e., no abnormal values) and adjusted for the following variables: maternal age, BMI, gestational week, parity, gravidity, weight gain, education, physical activity, smoking, drinking, delivery model, and fetal sex

^b^*P*<0.05 for the trend with the increased number of abnormal OGTT values

#### The risk of elevated TSH status based on the type and number of abnormal OGTT values during pregnancy

The plotted spline curves showed an upward trend in the incidence of elevated TSH status as OGTT values increased, particularly for fasting and 1-hour glucose (Fig. 1d and i). These findings were supported by both crude and adjusted linear regression analyses (Table S1). Among newborns of women without gestational diabetes mellitus (GDM) (zero abnormal OGTT value), the incidence of elevated TSH status (TSH > 10 mIU/L) in the offspring was 6.3 per 1,000 live births. In contrast, newborns exposed to women with three abnormal values had an almost five-fold higher risk of elevated TSH status (incidence rate 26.7 per 1,000 live births; adjusted RR 4.77 [95% CI (1.64–13.96]). For newborns of women with GDM, the risk of elevated TSH status (TSH > 20 mIU/L) increased with the number of abnormal OGTT values (Table 3).

**Table 3:** Risk of elevated TSH status in the offspring by number and type of abnormal OGTT values (*N* = 15,236)

Abnormal OGTT value	TSH > 10mIU/L			TSH > 20mIU/L		
	Case (incidence rate, %)	Crude RR (95% CI)	Adjusted RR^a^ (95% CI)	Case (incidence rate, %)	Crude RR (95% CI)	Adjusted RR^a^ (95% CI)
*Abnormal values, n*						
0	86(0.63)	Reference	Reference	21(0.15)	Reference	Reference
≥ 1 (GDM)	12(0.68)	1.07(0.59–1.96)	1.13(0.60–2.12)	5(0.29)	1.83(0.69–4.85)	2.14(0.76–6.01)
1	6(0.49)	0.76(0.33–1.73)	0.80(0.34–1.85)	2 (0.16)	1.04(0.24–4.43)	1.20(0.31–17.71)
2	2(0.54)	0.86(0.21–3.48)	0.96(0.23–3.98)	1(0.27)	1.76(0.24–13.04)	2.36(0.13–11.80)
3	4(2.67)	**4.12(1.53–11.09**)	**4.77(1.64–13.96)**	**2(1.33)**	**8.44(2.00-35.68)** ^**b**^	**12.76(2.29–71.18)** ^**b**^
*Abnormal values, type*						
Fasting (≥ 5.1 mmol/L)	8(0.97)	1.56(0.75–3.20)	1.59(0.76–3.35)	5(0.60)	**4.17(1.58–11.02)**	**4.48(1.57–12.73)**
1-hour (≥ 10.0 mmol/L)	7(0.97)	1.56(0.72–3.35)	1.69(0.77–3.74)	3(0.42)	2.64(0.79–8.78)	3.34(0.99–11.32)
2-hour (≥ 8.5 mmol/L)	7(0.79)	1.25(0.58–2.69)	1.39(0.63–3.04)	2(0.23)	1.36(0.32–5.73)	1.76(0.40–7.76)

Bold font indicates significant association. OGTT, oral glucose tolerance test results; GDM, gestational diabetes mellitus; TSH, thyroid stimulating hormone; RR, risk ratio; CI, confidence interval

^a^Values reflect the results of modified Poisson regression model including women without GDM as the reference group (i.e., no abnormal values) and adjusted for the following variables: maternal age, BMI, gestational week, parity, gravidity, weight gain, education, physical activity, smoking, drinking, delivery model, and fetal sex

^b^*P*<0.05 for the trend with the increased number of abnormal OGTT values

Newborns exposed to women with three abnormal values also had a higher risk of elevated TSH status (TSH > 20 mIU/L) (adjusted RR, 12.76 [95% CI 2.29–71.18]). Moreover, newborns of women with abnormal fasting blood glucose had a significantly elevated risk of elevated TSH status in both crude (4.17, 95%CI: 1.58–11.02) and adjusted models (4.48, 95%CI: 1.57–12.73), consistent with the second prediction model (Table 4). However, other numbers or types of abnormal OGTT values were not associated with elevated TSH status risk, even in the second prediction model that included all three OGTT values.

**Table 4:** Independent association of abnormal types of fasting, 1-h, and 2-h OGTT values with TSH status in the offspring (*N* = 15,236)

Abnormal values,type	TSH > 10mIU/L		TSH > 20mIU/L	
	Crude RR (95% CI)	Adjusted RR^a^ (95% CI)	Crude RR (95% CI)	Adjusted RR^a^ (95% CI)
Fasting (≥ 5.1 mmol/L)	1.42(0.72–2.77)	1.42(0.72–2.80)	**3.83(1.60–9.19)**	**3.82(1.55–9.42)**
1-hour (≥ 10.0 mmol/L)	1.40(0.68–2.86)	1.47(0.70–3.05)	1.91(0.71–5.16)	2.21(0.84–5.79)
2-hour (≥ 8.5 mmol/L)	0.99(0.47–2.11)	1.09(0.51–2.34)	0.62(0.17–2.27)	0.81(0.21–3.07)

Bold font indicates significant association. OGTT, oral glucose tolerance test results; TSH, thyroid stimulating hormone; RR, risk ratio; CI, confidence interval

^a^Results of modified Poisson regression model, adjusted for all three OGTT values as well as maternal age, BMI, gestational week, parity, gravidity, weight gain, education, physical activity, smoking, drinking, delivery model, and fetal sex. The reference group for each of the variables is the group of women in whom the corresponding OGTT value was within normal limits

### Additional analyses

After stratifying analyses by offspring sex and maternal age, no consistent heterogeneity was found for the association between OGTT results and offspring TSH levels. By contrast, associations between OGTT results and offspring TSH levels appeared stronger among females with overweight (Table S3-S5). All sensitivity analyses yielded consistent results with the main analyses (data not shown).

## Discussion

This large birth cohort study provides innovative insights as it is the first study to examine the risk of elevated TSH status in offspring of women with GDM based on the type and number of abnormal OGTT values during pregnancy. Our main findings were that among women with GDM, (1) an increase in OGTT values was associated with a considerable increase in the neonatal TSH levels; (2) the risk of elevated TSH status in offspring seemed to be greatest for women with three abnormal glucose values; (3) maternal fasting blood glucose was independently positively associated with neonatal TSH levels and risk of elevated TSH status.

Although many studies have reported an association between maternal thyroid function and GDM [23–25], few have specifically examined the effects of GDM even OGTT results on offspring thyroid function. The current large prospective study provided comprehensive and consistent evidence for the association between OGTT results and offspring TSH levels. Several studies have shown no significant association between GDM and umbilical cord TSH concentration [26–29]. However, the limitations of these studies are the lack of adjustment for potential confounding factors. In our study, we found that offspring exposed to women with GDM had higher TSH levels after adjustment for various confounders. Consistent with our results, a study in China found that offspring TSH levels were significantly increased in the uncontrolled GDM group compared with the normal and controlled GDM groups, whereas the levels of triiodothyronine (T3), T4 were significantly decreased. Another case-control study involving 469 diet-controlled GDM pregnancies and 474 non-diabetic pregnancies found that women with GDM had higher cord blood TSH concentrations. Additionally, they also found a positive correlation between TSH levels and OGTT 2 h values [10]. While our study results indicated a significant correlation between fasting blood glucose and TSH levels, differences in population characteristics and sample size may partially explain the discrepancies in results. Interestingly, studies linking GDM with elevated TSH levels have been exclusively conducted in Asian countries. This could be attributed to the relatively higher levels of cord blood TSH levels in individuals with Asian ancestry compared to other ancestries [28, 30]. Further research is needed to investigate this issue in different racial and ethnic groups.

In our study, although no significant association was detected between GDM and elevated TSH status, the offspring of women with three abnormal glucose values showed a greater risk of elevated TSH status. A study also identified an increased incidence of neonatal hyperthyrotropinemia (TSH > 16 mIU/L) associated with GDM, which is in line with our results [10]. Subsequently, the same research group published an article revealing that increased TSH levels were associated with fetal intrauterine stress caused by GDM after adjusting for confounding factors [31]. However, these two studies were published twenty years ago and additional research on this topic has been limited in recent years. Therefore, our findings should be validated in future studies and further evidence on the association between GDM and elevated TSH status is also needed.

This study reveals that maternal fasting glucose levels are more strongly associated with neonatal TSH levels and the risk of elevated TSH status in offspring than postprandial glucose levels, suggesting that fasting glucose is a more critical predictor of GDM impacts. This aligns with previous research indicating that fasting glucose levels can forecast adverse pregnancy outcomes and are key indicators of metabolic disorders, hypertension, and other health issues [32–35]. These findings underscore the importance of focusing on fasting glucose levels in GDM screening and management, highlighting the potential need for different treatment approaches based on GDM subtypes.

To explore the potential biological mechanisms underlying the observed associations, several hypotheses could be considered. Previous study hypothesizes that maternal hyperglycemia might lead to thyroid dysfunction in the fetus by altering the expression of thyroid hormone-regulating genes and influencing T3 and T4 conversion [36, 37]. Additionally, maternal hyperglycemia might induce oxidative stress and inflammation in both maternal and fetal bodies, impacting thyroid function by impairing thyroid hormone utilization and increasing cytokines that disrupt hormone synthesis and metabolism [38–43]. Further research into these mechanisms could enhance our understanding of how maternal glycemic control affects fetal thyroid function and the risk of elevated TSH in offspring.

This study significantly contributes to clinical practice by highlighting the need for personalized management of pregnant women with GDM, underscoring the importance of stringent glycemic control not only for maternal health but also for preventing neonatal thyroid dysfunctions and potential long-term developmental issues in offspring. The study advocates for a multidisciplinary approach involving endocrinologists, obstetricians, and pediatricians to address the complex interplay between maternal glycemia and neonatal health. It underscores the importance of considering different types of glycemic anomalies in GDM (e.g., fasting vs. postprandial glucose levels) for targeted interventions. These insights are vital for shaping long-term health strategies, including public health policies and awareness programs, to mitigate the impact of GDM on future generations. Overall, this research provides a valuable framework for clinicians to develop more effective treatment and monitoring strategies, potentially leading to improved health outcomes for both mothers and their children.

Our study has significant strengths, such as a large sample size and a population-based design, facilitating the comprehensive capture of diverse covariate data. Additionally, the diagnosis of GDM was based on OGTT data rather than relying on the diagnostic codes from the International Classification of Diseases. We acknowledge several potential limitations. First, the absence of data on maternal thyroid function is notable, though the exclusion of women with pre-existing thyroid disorders mitigates potential bias. Second, despite extensive adjustments for covariates to account for the associations of interest, residual confounding factors, including insulin use and iodine status, might not have been completely addressed. Third, the low incidence rate of elevated TSH status limited the number of cases, hindering further stratified analyses. Fourth, due to the lack of follow-up data or specific diagnostics for CH, our results necessitate validation through future studies. Last, the restriction of our cohort study to specific clinical centers and regions may limit the generalizability of our findings to more diverse populations.

The study identifies several key areas for future investigation to address unresolved questions in the field. Firstly, there is a critical need to explore the impact of maternal thyroid function during pregnancy on neonatal TSH levels, given its potential significant influence. Longitudinal studies tracking the long-term neurodevelopmental outcomes of infants with elevated TSH levels are also essential to understand the full scope of this condition’s impact. Moreover, expanding research to include diverse populations across different races and regions is vital to establish the generalizability of the findings. Investigating the specific effects of different GDM subtypes, particularly in relation to fasting and postprandial glucose levels, on neonatal TSH levels could provide deeper insights into tailored management strategies for GDM. Additionally, research focusing on gestational lifestyle factors and their interplay with GDM and neonatal TSH levels could unveil new avenues for preventive measures. These areas of future research are crucial for developing a more comprehensive understanding of GDM’s impact on neonatal health and for devising effective strategies to mitigate its consequences.

## Conclusions

This prospective birth cohort study revealed a negative correlation between OGTT results and increased risk of elevated TSH in offspring. Specifically, fasting blood glucose levels during pregnancy were more indicative of the risk of elevated TSH status. These findings suggest that OGTT can be a critical tool for clinicians to predict and manage the risk of neonatal thyroid dysfunction, guiding the development of targeted treatments to prevent related developmental issues.

### Electronic supplementary material

Below is the link to the electronic supplementary material.

Supplementary Material 1

## Data Availability

The datasets used or analyzed during the current study are available from the corresponding author on reasonable request.

## Abbreviations

GDM: Gestational Diabetes Mellitus
OGTT: Oral Glucose Tolerance Test
IADPSG: International Association of Diabetes and Pregnancy Study Groups
CH: Congenital Hypothyroidism
TSH: Thyroid Stimulating Hormone
RR: Risk Ratio
OR: Odds Ratio
CI: Confidence Interval
T4: Thyroxine
T3: Triiodothyronine
