# Supplementary material for: Associations between OGTT results during pregnancy and offspring TSH levels: a birth cohort study
Source: BMC Pregnancy Childbirth. 2024 May 17;24:375. doi: 10.1186/s12884-024-06554-4 (PMC11100047; doi:10.1186/s12884-024-06554-4)
Supplement: Supplementary file 1 — Supplementary Material 1 [file 12884_2024_6554_MOESM1_ESM.docx]

**Associations between OGTT results during pregnancy and offspring TSH levels: A birth cohort study**

Meng Yang, MD^1^†, Zhongqiang Cao, MD^1^†, Jiayi Cheng, MSc^2^, Wanting Zhu, MSc^2^, Xiaoyuan Feng, MSc^3^, Jieqiong Zhou, MD^2^, Jiuying Liu, MD^2^, Yuanyuan Zhong, MD^2^, Yan Zhou, MD^2^, Hong Mei, MD^1^, Xiaonan Cai, MSc^1^, Liqin Hu, MD^1^, Aifen Zhou, MD^1*^, Han Xiao, MD^1*^

**Affiliations:**

^1^Institute of Maternal and Child Health, Wuhan Children’s Hospital (Wuhan Maternal and Child Health care Hospital), Tongji Medical College, Huazhong University of Science and Technology, Wuhan, China.

^2^Department of Obstetrics, Wuhan Children’s Hospital (Wuhan Maternal and Child Health care Hospital), Tongji Medical College, Huazhong University of Science and Technology, Wuhan, China.

^3^Department of echocardiography, Wuhan Children’s Hospital (Wuhan Maternal and Child Health care Hospital), Tongji Medical College, Huazhong University of Science and Technology, Wuhan, China.

**Short title:** OGTT results and TSH levels.

†These authors contributed equally to this article.

***Corresponding author:** Institute of Maternal and Child Health, Wuhan Children’s Hospital (Wuhan Maternal and Child Health care Hospital), Tongji Medical College, Huazhong University of Science and Technology, Wuhan 430000, China

Aifen Zhou, [april1972@163.com](mailto:april1972@163.com); Han Xiao, [tjxiaohan1980@163.com](mailto:tjxiaohan1980@163.com)


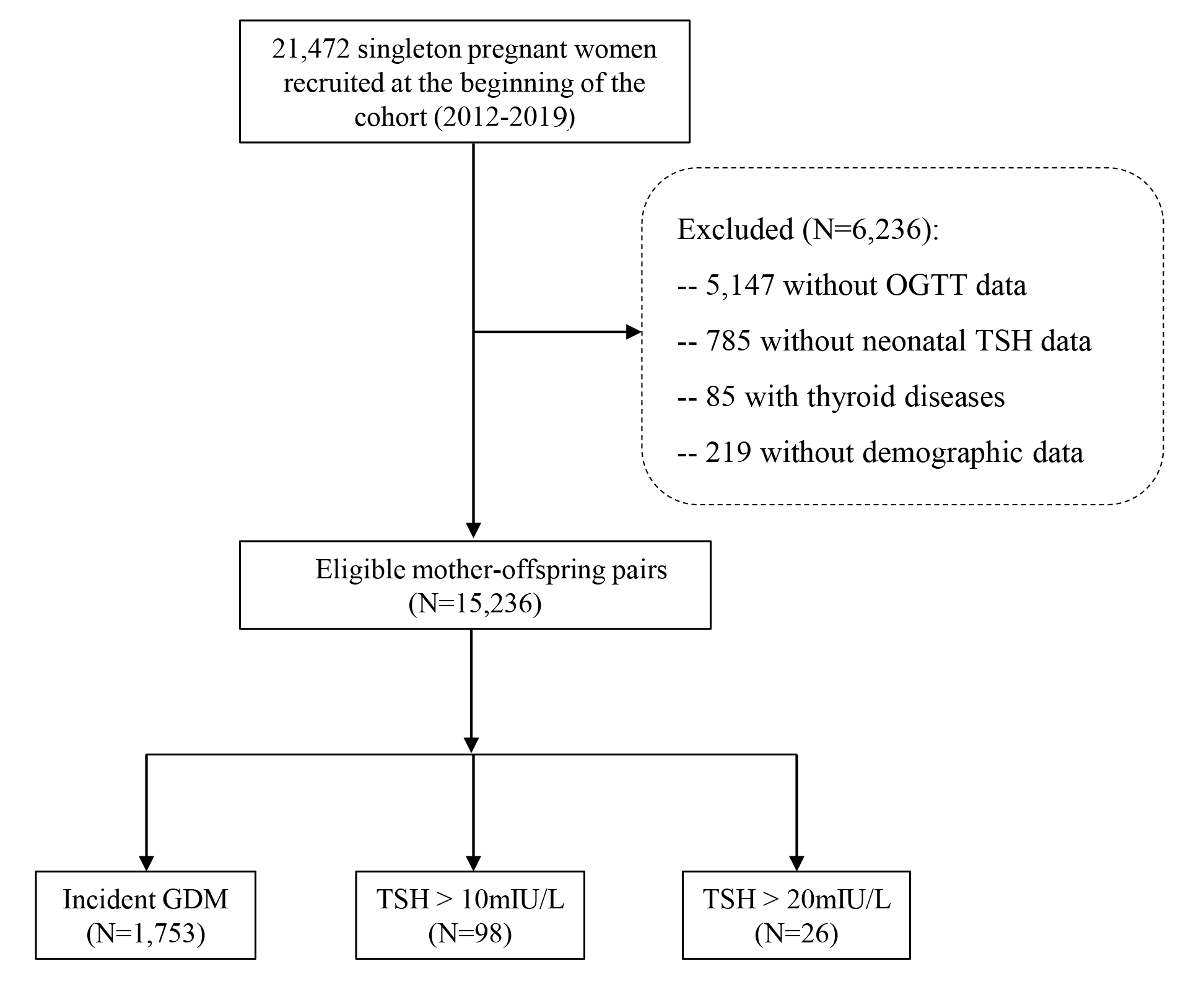


Figure S1. Study population flow diagram.

Table S1. Association of fasting, 1-h, and 2-h OGTT values with TSH levels and TSH status in the offspring.

| OGTT value | TSH (mIU/L) | |  |  | TSH>10mIU/L | |  | TSH>20mIU/L | |
| --- | --- | --- | --- | --- | --- | --- | --- | --- | --- |
|  | Crude β  (95% CI) | Adjusted β^a^  (95% CI) |  |  | Crude RR  (95% CI) | Adjusted RR^a^  (95% CI) |  | Crude RR  (95% CI) | Adjusted RR^a^  (95% CI) |
| Fasting (mmol/L) | 0.10(-0.01,0.20) | **0.11(0.01,0.22)** |  |  | **1.21(1.09-1.34)** | **1.25(1.12-1.39)** |  | **1.28(1.14-1.43)** | **1.32(1.16-1.51)** |
| 1-hour (mmol/L) | **0.04(0.01,0.07)** | **0.06(0.02,0.89)** |  |  | **1.17(1.06-1.29)** | **1.22(1.09-1.36)** |  | **1.22(1.02-1.48)** | **1.30(1.07-1.58)** |
| 2-hour (mmol/L) | 0.03(-0.01,0.06) | 0.03(-0.01,0.06) |  |  | **1.05(1.01-1.20)** | **1.07(1.02-1.11)** |  | 0.91(0.65-1.29) | 0.96(0.67-1.37) |

Bold font indicates significant association. OGTT, oral glucose tolerance test results; TSH, thyroid stimulating hormone; CH, congenital hypothyroidism.

^a^Results adjusted for maternal age, BMI, gestational week, parity, gravidity, weight gain, education, physical activity, smoking, drinking, delivery model, and fetal sex.

Table S2. Independent association of abnormal types of fasting, 1-h, and 2-h OGTT values with TSH levels in the offspring.

| **Abnormal values, type** | TSH (mIU/L) | |
| --- | --- | --- |
|  | Crude β (95% CI) | Adjusted β (95% CI)^a^ |
| Fasting (≥5.1 mmol/L) | **0.26(0.02,0.50)** | **0.27(0.03,0.51)** |
| 1-hour (≥10.0 mmol/L) | 0.08(-0.19,0.35) | 0.11(-0.17,0.38) |
| 2-hour (≥8.5 mmol/L) | -0.06(-0.31,0.18) | -0.01(-0.25,0.24) |

Bold font indicates significant association. OGTT, oral glucose tolerance test results; TSH, thyroid stimulating hormone; CI, confidence interval.

^a^Results of linear regression model, adjusted for all three OGTT values as well as maternal age, BMI, gestational week, parity, gravidity, weight gain, education, physical activity, smoking, drinking, delivery model, and fetal sex.

Table S3. Changes of TSH levels in the offspring by number and type of abnormal OGTT values, stratified by offspring sex (N=15,236).

| Abnormal OGTT value | Adjusted β^a^ (95% CI) | |
| --- | --- | --- |
|  | Male(n=8,066) | Female(n=7,255) |
| **Abnormal values, n** |  |  |
| 0 | Reference | Reference |
| ≥1 (GDM) | 0.15(-0.04,0.34) | 0.26(-0.01,0.53) |
| 1 | 0.16(-0.07,0.39) | 0.29(-0.02,0.60) |
| 2 | -0.03(-0.41,0.34) | 0.18(-0.42,0.78) |
| 3 | 0.61(-0.01,1.24) | 0.13(-0.72,0.98) |
| **Abnormal values, type** |  |  |
| Fasting (≥5.1 mmol/L) | **0.13(0.01,0.25)** | 0.09(-0.08,0.27) |
| 1-hour (≥10.0 mmol/L) | 0.06(0.02,0.09) | 0.05(-0.01,0.11) |
| 2-hour (≥8.5 mmol/L) | 0.05(0.01,0.10) | 0.03(-0.03,0.09) |

Bold font indicates significant association. OGTT, oral glucose tolerance test results; GDM, gestational diabetes mellitus; TSH, thyroid stimulating hormone.

^a^Values reflect the results of linear regression model including women without GDM as the reference group (i.e., no abnormal values) and adjusted for the following variables: maternal age, gestational week, BMI, parity, gravidity, weight gain, education, physical activity, smoking, drinking and delivery model.

^b^*P*<0.05 for the trend with the increased number of abnormal OGTT values.

Table S4. Changes of TSH levels in the offspring by number and type of abnormal OGTT values, stratified by maternal age (N=15,236).

| Abnormal OGTT value | Adjusted β^a^ (95% CI) | |
| --- | --- | --- |
|  | Age>30 (n=4,284) | Age<=30(n=10,952) |
| **Abnormal values, n** |  |  |
| 0 | Reference | Reference |
| ≥1 (GDM) | 0.19(0.01,0.36) | 0.20(-0.03,0.43) |
| 1 | 0.18(-0.03,0.39) | 0.24(-0.02,0.50) |
| 2 | 0.29(-0.04,0.63) | -0.15(-0.64,0.34) |
| 3 | -0.03(-0.51,0.46) | 0.79(-0.02,1.61) |
| **Abnormal values, type** |  |  |
| Fasting (≥5.1 mmol/L) | **0.20(0.05,0.34)** | -0.03(-0.14,0.08) |
| 1-hour (≥10.0 mmol/L) | **0.06(0.02,0.10)** | **0.05(0.01,0.10)** |
| 2-hour (≥8.5 mmol/L) | **0.04(0.01,0.08)** | 0.04(-0.01,0.10) |

Bold font indicates significant association. OGTT, oral glucose tolerance test results; GDM, gestational diabetes mellitus; TSH, thyroid stimulating hormone.

^a^Values reflect the results of linear regression model including women without GDM as the reference group (i.e., no abnormal values) and adjusted for the following variables: gestational week, BMI, parity, gravidity, weight gain, education, physical activity, smoking, drinking, delivery model, and fetal sex.

Table S5. Changes of TSH levels in the offspring by number and type of abnormal OGTT values, stratified by maternal BMI (N=15,236).

| Abnormal OGTT value | Adjusted β^a^ (95% CI) | |
| --- | --- | --- |
|  | BMI>=24 (n=2,066) | BMI<24(n=1,3170) |
| **Abnormal values, n** |  |  |
| 0 | Reference | Reference |
| ≥1 (GDM) | **0.61(0.14,1.07)** | 0.11(-0.07,0.28) |
| 1 | **0.80(0.24,1.35)** | 0.09(-0.11,0.29) |
| 2 | 0.15(-0.72,1.03) | 0.04(-0.33,0.41) |
| 3 | 0.46(-0.61,1.52) | 0.47(-0.18,1.11) |
| **Abnormal values, type** |  |  |
| Fasting (≥5.1 mmol/L) | 0.18(-0.12,0.48) | 0.10(-0.01,0.22) |
| 1-hour (≥10.0 mmol/L) | **0.05(0.02,0.09)** | 0.07(-0.04,0.18) |
| 2-hour (≥8.5 mmol/L) | **0.06(0.02,0.09)** | -0.04(-0.16,0.09) |

Bold font indicates significant association. OGTT, oral glucose tolerance test results; GDM, gestational diabetes mellitus; TSH, thyroid stimulating hormone.

^a^Values reflect the results of linear regression model including women without GDM as the reference group (i.e., no abnormal values) and adjusted for the following variables: maternal age, gestational week, parity, gravidity, weight gain, education, physical activity, smoking, drinking, delivery model, and fetal sex.
